# Exploring positive teacher-student relationships: the synergy of teacher mindfulness and emotional intelligence

**DOI:** 10.3389/fpsyg.2023.1301786

**Published:** 2023-11-29

**Authors:** Xue Wang

**Affiliations:** Department of Foreign Languages, Henan Finance University, Zhengzhou, Henan, China

**Keywords:** teacher mindfulness, teacher-student relationship quality, emotional intelligence, English teachers, structural equation modeling

## Abstract

**Introduction:**

This study delves into the intricate synergy between teacher mindfulness and the quality of teacher-student relationships, with a specific and deliberate focus on the mediating influence of emotional intelligence. The way teachers engage with their students not only impacts the learning outcomes but also contributes significantly to the overall classroom atmosphere. Understanding the underlying mechanisms that drive these relationships is crucial for educators and policymakers alike. This research seeks to shed light on these critical dynamics.

**Methods:**

To investigate this multifaceted interplay, a participant pool of 369 Chinese English teachers was assembled. The research employed a comprehensive approach to data collection, utilizing self-report questionnaires completed by the instructors. Structural equation modeling, a robust statistical technique, was employed to rigorously analyze the collected data.

**Results:**

The data analysis unveiled a robust and direct association between teacher mindfulness and the quality of teacher-student relationships. Beyond this primary link, a noteworthy revelation emerged: emotional intelligence, as measured through our analysis, was identified as a pivotal mediating factor in this relationship. This finding highlights the intricate web of emotions, awareness, and interpersonal interactions that underpin effective teaching and positive teacher-student relationships.

**Discussion:**

These significant findings underscore the critical roles of teacher mindfulness and emotional intelligence in shaping the educational landscape. The implications of this study reach far beyond academia, extending to the development of tailored educational interventions and support strategies.

## Introduction

Individuals’ desire to feel linked to people is an essential psychological desire, and the need to develop social relationships is one of the strongest human motivations (Baumeister and Leary, 1995). The urge for relatedness, or the sense of connection to others, may be a strong motivator for behavior that supports interpersonal connections in educational settings (Walton et al., 2012). In the past few years, research on the importance of strong teacher-student relationships has received growing attention (Roorda et al., 2011; Longobardi et al., 2021; Meng, 2021; Derakhshan, 2022b; Fabris et al., 2023; Forsberg et al., 2023). Pupils are better able to handle the cognitive and emotional responsibilities of school when their requirements for relatedness are met (Martin and Dowson, 2009), and the long-term learners’ education is established steadily through strong teacher-student relationships (Hamre and Pianta, 2001). When students believe their professors like them, they will do better academically and have more school dedication (Wang and Eccles, 2013; Longobardi et al., 2016; Henry and Thorsen, 2018). Furthermore, a multitude of research has demonstrated that students who foster close connections with their teachers tend to exhibit higher levels of academic interest, dedication, achievement, self-efficacy, and motivation compared to those with more distant relationships (Sakiz et al., 2012; Tosto et al., 2016; Henry and Thorsen, 2018; de Ruiter et al., 2021; Meng, 2021; Fabris et al., 2022; Longobardi et al., 2023). Conversely, when students perceive a lack of support from their teachers, it often results in higher rates of class absenteeism; however, on the contrary, when they experience a strong sense of teacher-student rapport or trust, they are more motivated to attend their classes (Demir et al., 2019). Also, when students regularly attend class, they have more chances to get to know their teachers, which may encourage ongoing engagement, and learning transfers are also aided by strong teacher-student relationships (Cheung, 2019). Learners adjusted to school easier when they were engaged and confident in their abilities, and when they worked with their teachers more frequently, they showed higher levels of inspiration (Liu and Chiang, 2019). Generally, favorable teacher-student connections are directly associated with academic competence and accomplishment, and these connections have favorable implications for scholarly outcomes (Valiente et al., 2008; Wubbels et al., 2016).

The second variable examined in this study is teacher mindfulness. Mindfulness plays a pivotal role in assisting educators, particularly in navigating challenging circumstances. It enables teachers to maintain a heightened level of focus, a deep understanding of the learning process, and a nuanced awareness of classroom dynamics. These qualities, as research suggests, can lead to a reduction in teachers’ stress and despair, consequently enhancing their interactions with students (Becker et al., 2017; Wang et al., 2023). Moreover, according to Braun et al. (2019), mindfulness emerges as a robust predictor of instructors’ professional well-being and their capacity for effective classroom management. However, mindfulness is not limited to personal attributes alone; it also encompasses the enduring practice of acceptance. This practice fosters a positive emotional climate, with students perceiving warmth and positivity from their teachers. Teachers who embody this quality are naturally more attuned to their students’ needs, treating them with greater tolerance. As a result, this dynamic contributes to the cultivation of positive teacher-student relationships (Frank et al., 2016; Longobardi et al., 2021).

Since numerous studies have emphasized instructors’ cooperation with students as a noticeable component in establishing a crucial base and inspiration for the teaching and learning process, relationships between both teachers and students affect learning circumstances. Furthermore, emotional intelligence, which refers to the capacity to recognize, comprehend, and regulate emotions within oneself and others (Salovey and Mayer, 1990), represents another crucial aspect that could potentially act as a mediator in the association between teacher mindfulness and teacher-student relationship quality. Educators with elevated emotional intelligence may demonstrate a heightened awareness of their students’ emotional states, fostering increased empathy and attentiveness during their interactions (Pozo-Rico and Sandoval, 2020; Valente and Lourenço, 2020). Emotional intelligence has been linked to improved teacher-student relationships (Chamizo-Nieto et al., 2021), as instructors with greater emotional intelligence may better navigate conflicts and provide appropriate emotional support to their students (Mérida-López and Extremera, 2017; Wang et al., 2023).

While both teacher mindfulness and emotional intelligence have been previously associated with fostering positive teacher-student relationships, the intricate interplay between these two factors, as well as the potential mediating role of emotional intelligence, remains an underexplored area in the current body of literature. Therefore, the primary objective of the present study is to bridge this research gap by conducting a thorough investigation into the relationship between these variables. We will place particular emphasis on the potential mediating function of emotional intelligence within a sample comprising Chinese English teachers. By delving into the intricate dynamics among teacher mindfulness, emotional intelligence, and the quality of teacher-student relationships, our research seeks to significantly enhance our understanding of the mechanisms that underpin positive teacher-student interactions. The implications of our findings extend beyond mere academic curiosity. They hold the potential to provide valuable insights for the design and implementation of targeted educational interventions and support strategies. These strategies are envisioned to not only nurture the development of mindfulness and emotional intelligence among teachers but also to create an environment conducive to enriched teacher-student relationships. Such a transformation in the teacher-student dynamic is expected to have a profound, positive impact on the overall dynamics of the classroom.

## Literature review

### Mindfulness

Although the concept of mindfulness is neither intrinsically spiritual nor religious, the first accounts of it came from Buddhist teachings (Olendzki, 2005). Eastern Buddhism is where mindfulness initially emerged, and Western psychologists later adapted it to psychology to help people deal with their negative feelings as well as their stress (Grabovac et al., 2011; Wang et al., 2023). According to Brown and Ryan (2003), the term “mindfulness” is related to an extremely focused attention state in which people keep their concentration on an ongoing incident or situation and embrace it without reservation. Mindful people perceive, narrate, assess, and accept what they have experienced without making judgments (Kabat-Zinn, 1994). According to Kabat-Zinn (1990), mindfulness entails being receptive to new experiences and capable of experiencing them in the here and now. It is also linked to the capacity to let go of the past and not cling to ideas or feelings (Bishop et al., 2004). By separating the ego from an event and observing current internal and external stimuli logically, mindfulness enables people to lessen their propensity for depression and psychological suffering (Mesmer-Magnus et al., 2017). In essence, when one is conscious, they perceive reality rather than an idealized version of it. Therefore, consciousness in the current moment and nonjudgmental cognitive breadth are the distinguishing qualities of mindfulness (Shapiro et al., 2006; Dane, 2011; Keng et al., 2011).

Mindfulness has emerged as a prominent construct in various fields, including psychology, education, and well-being, garnering extensive attention for its potential benefits in promoting positive outcomes across diverse contexts (Langer, 1989; Fathi et al., 2023). Originally rooted in Eastern contemplative practices, mindfulness has been adapted and integrated into contemporary research and interventions, offering a unique lens through which to examine human cognition, emotions, and behavior (Bishop et al., 2004). Within the realm of education, mindfulness has become a subject of growing interest, particularly in understanding its implications for teachers and the teacher-student relationship (Schonert-Reichl and Roeser, 2016).

Furthermore, recent studies have significantly advanced our understanding of the interplay between mindfulness, emotional intelligence, and their positive outcomes in educational settings. It is essential to recognize that mindfulness is intricately associated with emotional intelligence (Galloway et al., 2022). These two constructs share common elements, and when combined, they have a profound impact on the emotional learning of learners. Maloney et al. (2016) exemplify the potential of mindfulness in enhancing emotional intelligence among school-aged children, thereby contributing to their overall well-being. Furthermore, Galloway et al. (2022) demonstrate the synergistic effects of mindfulness and emotional intelligence, shedding light on the broader implications of these constructs in the context of couple relationship satisfaction.

Mindfulness involves a heightened level of conscious awareness, allowing individuals to be fully present and accepting of their experiences without attachment or reactivity (Keng et al., 2011). Research has indicated that mindfulness practices and interventions can lead to various positive outcomes, such as reduced stress and burnout (Luken and Sammons, 2016), improved emotional regulation (Hill and Updegraff, 2012), and enhanced overall well-being (Shapiro et al., 2006). In the context of education, studies have begun to explore the role of teacher mindfulness and its potential impact on teacher-student interactions and classroom dynamics. Teachers who cultivate mindfulness may exhibit increased emotional regulation, reduced stress, and improved empathy, which can contribute to more positive teacher-student relationships (Roeser et al., 2012; Braun et al., 2019). Mindful teachers are believed to be better equipped to respond to students’ emotional needs, navigate conflicts constructively, and create a nurturing and supportive learning environment (MacDonald and Shirley, 2009; Moyano et al., 2021).

Notably, empirical evidence regarding the relationship between teacher mindfulness and teacher-student relationship quality is still emerging and warrants further investigation (Cochran and Peters, 2023; Yuan et al., 2023). Some studies have shown positive associations between teacher mindfulness and positive student outcomes, such as increased student engagement and decreased disruptive behaviors (Emerson et al., 2017; Jennings and DeMauro, 2017). However, additional research is needed to provide a comprehensive understanding of the mechanisms through which teacher mindfulness may influence teacher-student relationships. One potential mechanism that may explain the link between teacher mindfulness and teacher-student relationship quality is emotional intelligence. Teachers with high emotional intelligence are better positioned to empathize with their students’ experiences, build trust, and foster positive emotional connections (Mérida-López and Extremera, 2017). Emotional intelligence has been found to contribute significantly to teacher-student relationship quality, supporting the idea that it may serve as a mediator in the relationship between teacher mindfulness and teacher-student interactions (Wang et al., 2023).

To bridge the gap in the literature and enhance our understanding of the interplay between teacher mindfulness, emotional intelligence, and teacher-student relationship quality, the present study endeavors to explore these relationships among Chinese English teachers. By shedding light on the potential mediating role of emotional intelligence, this research aims to contribute valuable insights to the existing body of knowledge, informing educational interventions and support strategies that promote mindful teaching practices and nurturing teacher-student relationships.

### Teacher-student relationship

Relationships between instructors and students that are highly sensitive, friendly, and honest have been described as having “teacher-student rapport” (Frymier and Houser, 2000; Frisby and Martin, 2010), and it addresses “the degree to which students feel supported, respected, and valued by their teachers” (Piechurska-Kuciel, 2011, p. 85). Therefore, in order to build a solid connection with pupils, instructors should continuously encourage them, respect their opinions, and recognize their academic work (Schriver and Harr Kulynych, 2021). Accordingly, Thornberg et al. (2020) made the case that instructors might develop close bonds with their pupils by upholding their academic standards. Thompson (2018) also argued that instructors can only develop a friendly, mutually beneficial connection with their students if they show concern for their requirements and passions. According to Virat (2022), exhibiting empathy and understanding aids instructors in creating a psychological connection with their pupils. In educational settings characterized by strong teacher-student relationships, learners frequently invest more time and effort in completing their academic tasks (Roorda et al., 2011; Pianta et al., 2012; Quin, 2017; Longobardi et al., 2023; Wang and Derakhshan, 2023). According to Roorda et al. (2017), an emotionally charged teacher-student interaction is crucial for learners to succeed academically because it encourages them to take an active role in their learning environments.

The teacher-student relationship is universally acknowledged as a critical factor with profound implications for students’ academic, social, and emotional development (Hamre and Pianta, 2005; Roorda et al., 2011; Longobardi et al., 2016; Shakki, 2022; Fabris et al., 2023). A positive teacher-student relationship is characterized by fundamental elements such as trust, mutual respect, emotional support, and effective communication (Hamre et al., 2013). Conversely, negative or strained relationships can contribute to student disengagement, academic underperformance, and behavioral challenges (Baker et al., 2008). Extensive research in the realm of educational psychology and related fields underscores the profound significance of positive teacher-student relationships in shaping students’ well-being and academic outcomes (Hughes et al., 2012). Studies consistently demonstrate that students who experience positive relationships with their teachers are more likely to exhibit heightened levels of engagement, motivation, and a genuine enthusiasm for learning (Froiland et al., 2019; Howe et al., 2019). Moreover, such positive teacher-student relationships have been associated with increased student self-esteem, a profound sense of belonging, and a heightened overall satisfaction with their school experience (Fauth et al., 2019; Liebowitz and Porter, 2019; Longobardi et al., 2021).

The quality of the teacher-student relationship is a multifaceted construct, comprising various dimensions that reflect the nature of the interactions between teachers and their students. One widely used measure to assess teacher-student relationship quality is the Teacher-Student Relationship Scale (STRS) developed by Pianta and Steinberg (1992). The STRS comprises three sub-scales: closeness, conflict, and dependency. The closeness sub-scale reflects the level of emotional connection and warmth between teachers and students. High scores on this sub-scale indicate positive relationships characterized by open communication and mutual affection. On the other hand, the conflict sub-scale measures the presence of negative interactions, tension, and disagreements in the teacher-student relationship. Lower scores on this sub-scale indicate a positive relationship with minimal conflict. Lastly, the dependency sub-scale assesses the extent to which students rely on their teachers for emotional support and guidance (Birch and Ladd, 1997).

Scholars have been consistently intrigued by the impact of teacher-student relationships on educational outcomes due to the fundamental relevance of these relationships in educational contexts (Wanders et al., 2020; Engels et al., 2021; Derakhshan, 2022a; Zhang et al., 2023). To determine if it influences Polish and Iranian students’ interest in achieving educational objectives, Derakhshan et al. (2022) investigated teacher-student rapport. To achieve this, 431 undergraduates were given two pre-made surveys. They discovered that the level of student involvement in achieving educational goals was positively predicted by both students’ teacher-student rapport. Similarly, Wanders et al. (2020) examined the effect of teacher-student rapport on pupils’ participation in the classroom with the help of 4,128 students who participated in two reliable surveys for this purpose. Data analysis revealed that teacher-student rapport can significantly influence pupil engagement in educational environments. Besides, Ibarra (2014) investigated how student-teacher interactions affect how actively students participate in class. Two pre-made scales were distributed among the 64 pupils in order to achieve this. The study of the students’ responses revealed that a positive teacher-student connection can boost students’ engagement in learning environments. Additionally, Engels et al. (2021) investigated the impact of emotional teacher-student connections on students’ school involvement in research as well. In doing so, a sizable sample of 5,382 pupils were given the questionnaires about teacher-student relationships and student involvement in school. Based on the comments they received, the investigators concluded that emotional teacher-student connections can have a significant impact on how engaged students are in their studies.

Numerous studies have established the impact of teacher-student relationship quality on various student outcomes. For example, a study by Hamre and Pianta (2001) demonstrated that high-quality teacher-student relationships were associated with greater student engagement, higher academic achievement, and decreased behavioral problems. Similarly, Roorda et al. (2011) found that positive teacher-student relationships were linked to improved student motivation and reduced school dropout rates. Furthermore, research has shown that positive teacher-student relationships can buffer the adverse effects of stress and support students’ resilience in challenging academic environments (Hughes et al., 2012).

Given the significance of teacher-student relationship quality in shaping students’ educational experiences and outcomes, it is essential to explore the factors that contribute to the establishment and maintenance of positive relationships. One potential factor that has gained attention in recent years is teacher mindfulness. Mindfulness practices and interventions have been associated with enhanced emotional regulation, increased empathy, and improved interpersonal relationships (Shapiro et al., 2006; Emerson et al., 2017).

According to attachment theory, the relationship between teachers and students is a continuation of the parent–child relationship and reflects the emotional bond between instructors and students, and it is also seen as a generally positive relationship state (Bergin and Bergin, 2009; Fitton, 2013; Fearon and Roisman, 2017). Student’s future educational achievement and adaptability will also be impacted by this association, in addition to their psychological, physical, interpersonal, and social growth (Rucinski et al., 2018; Camodeca and Coppola, 2019). The instructor takes the lead in the connection between the student and the teacher, and as a result, some teacher traits will have an impact on how well the parent-teacher connection works (Gillespie, 2005; Prewett et al., 2019). For instance, according to Becker’s et al. (2017) research, instructors need to maintain a high degree of emotional control and mental health in order to foster positive teacher-child relationships. In challenging circumstances, mindfulness can help instructors sustain a higher level of focus as well as knowledge of the learning process and classroom dynamics. By doing this, instructors can lower their levels of stress and despair, which can improve their interactions with their students (Becker et al., 2017; Burgos-Julián et al., 2022). Additionally, according to Braun et al. (2019), mindfulness can strongly predict instructors’ professional wellness and the quality of classroom management. However, mindfulness is also marked by persistent compassion. Students will experience favorable feelings from instructors as a result of them upholding this quality, which will make them more attentive to their requirements and treat them with greater tolerance (Frank et al., 2016; Lemberger-Truelove et al., 2018). Overall, investigating the relationship between teacher mindfulness and teacher-student relationship quality could provide valuable insights into how mindful teaching practices may contribute to fostering positive teacher-student interactions and supporting students’ overall well-being and success.

### Emotional intelligence

Emotional intelligence (EI) is a construct that encompasses the ability to perceive, understand, and manage one’s emotions effectively, as well as the capacity to recognize and respond empathetically to the emotions of others (Salovey and Mayer, 1990; Mayer et al., 2007). The ability to prioritize cognitive tasks that will benefit their present emotional state and adjust their feelings to better fit the scenario is a trait shared by those with strong emotional intelligence (Fiori and Vesely-Maillefer, 2018; Vesely Maillefer et al., 2018; Vesely-Maillefer and Saklofske, 2018). The concept of EI has garnered significant interest across multiple domains, encompassing psychology, education, and workplace contexts, owing to its potential implications for individual well-being, interpersonal relationships, and overall success (Brackett et al., 2011; Shakki, 2023).

Research on emotional intelligence has revealed its relevance in numerous aspects of individuals’ lives, particularly in the context of educational settings. In the realm of teaching, emotional intelligence is considered crucial for fostering positive teacher-student relationships and creating a supportive and nurturing learning environment (MacCann et al., 2020; Sánchez-Álvarez et al., 2020). Instructors with greater EI tend to exhibit greater empathy, understanding, and responsiveness to their students’ emotional needs, which can lead to increased student engagement and a sense of belonging in the classroom (Mortiboys, 2013; Keefer et al., 2018). Also, research has shown that teachers with greater emotional intelligence tend to have lower levels of burnout and stress, which can positively influence their job satisfaction and overall well-being (Mérida-López and Extremera, 2017; Greenier et al., 2021; Derakhshan et al., 2023; Geraci et al., 2023). The impact of teacher emotional intelligence on student outcomes has also been investigated in several studies. Teachers’ emotional intelligence has been associated with improved student academic achievement, social–emotional development, and overall school climate (Corcoran and Tormey, 2013; Pozo-Rico and Sandoval, 2020). Furthermore, teachers who are emotionally intelligent are better equipped to handle challenging situations and provide appropriate support to students experiencing emotional difficulties (Valente and Lourenço, 2020).

EI had already received a lot of consideration across all fields of psychology before the emergence of PP at the beginning of the 21st century (Salovey and Mayer, 1990; Mayer and Salovey, 1997; Seligman and Csikszentmihalyi, 2000). Since human productivity and general well-being are the main focuses of PP, EI has become increasingly popular, especially in the field of PP. Therefore, it is appropriate to include EI as a central idea of character qualities within PP (Salovey et al., 2002; Allen et al., 2014). A number of EI-related studies have been discovered as well and empirically demonstrated its crucial importance for language students as well as instructors (Shao et al., 2013; Mercer and Gkonou, 2017; Dewaele et al., 2018; Li, 2020; Fathi et al., 2021; Xiyun et al., 2022). As previously described, due to its close relationship with feelings, particularly good emotions (Zhang et al., 2022), EI is a key predictor of wellbeing (Nelis et al., 2011), and to be more precise, earlier research showed a negative correlation between EI and unfavorable emotions, specifically language anxiety. For instance, Dewaele et al. (2008) discovered that multilingual individuals with greater EI had a tendency to feel less nervous. According to Oxford (2017), those with greater EI viewed themselves as better capable of sensing and comprehending the feelings of their peers, controlling their own tension, and experiencing more confidence. According to Shao et al. (2013), in a Chinese educational setting, individuals who were higher in emotional intelligence often had lower levels of FLA, corroborating the association between EI and unfavorable second language classroom feelings.

Relevant research has illuminated the positive impact of mindfulness practice on the development of emotional abilities. For instance, Tomlinson et al. (2018) have highlighted a noteworthy negative correlation between mindfulness and various psychological issues, including depression, anxiety, tension, and emotional distress. Additionally, studies have revealed that individuals with higher levels of mindfulness exhibit an improved capacity to recognize, comprehend, and endure unpleasant emotions within their current circumstances (Osborne and Atkinson, 2022; Ainsworth et al., 2023; Wang et al., 2023). An individual’s connection with their feelings may be improved with the aid of mindfulness practice, which teaches people to actively sense both their own and other people’s feelings and view them from an “accepting” perspective (Chambers et al., 2009; Guendelman et al., 2017). The separation between them can aid people in improving their capacity for adaptable emotional function and emotional intelligence. According to Mayer’s et al. (2007) ability model, the capacity to identify, utilize, comprehend, and manage emotions is a component of emotional intelligence.

Given the importance of EI in the teaching profession and its potential implications for teacher-student interactions and student outcomes, understanding the role of emotional intelligence in affecting the link between teacher mindfulness and teacher-student relationship quality can provide valuable insights into the mechanisms through which EI influences the dynamics of teacher-student interactions and contributes to positive educational outcomes. Therefore, investigating the role of EI in the relationship between teacher mindfulness and teacher-student relationship quality can shed light on the potential benefits of cultivating emotional intelligence among teachers to enhance their effectiveness in fostering positive and supportive teacher-student relationships.

### The current research

The present study proposes a hypothesized model to explore the relationships between teacher mindfulness, teacher-student relationship quality, and the mediating role of emotional intelligence among English teachers. The conceptual framework is grounded in existing literature on mindfulness, emotional intelligence, and teacher-student relationships, providing a basis for the formulation of specific hypotheses.

*H*1: Drawing on prior investigations indicating a favorable correlation between teacher mindfulness and teacher-student relationship quality (Braun et al., 2019; Moyano et al., 2021; Wang et al., 2023), our hypothesis posits a meaningful and direct link between teacher mindfulness and teacher-student relationship quality. Specifically, we expect that teachers who demonstrate higher levels of mindfulness will have more positive and supportive relationships with their students, characterized by greater closeness, lower conflict, and reduced dependency on the teacher for emotional support.

*H*2: Given the well-established link between emotional intelligence and teacher-student relationship quality (Poulou, 2017; Chamizo-Nieto et al., 2021), we propose that emotional intelligence will mediate the relationship between teacher mindfulness and teacher-student relationship quality. Building upon the conceptual understanding of emotional intelligence as a capacity to understand and manage emotions, we posit that teachers with higher mindfulness may exhibit enhanced emotional intelligence, leading to more effective recognition and regulation of their own emotions and the emotions of their students. Consequently, teachers with greater emotional intelligence are expected to facilitate stronger and more positive teacher-student relationships, characterized by increased emotional understanding, empathy, and responsiveness. In other words, teachers’ mindfulness is predicted to contribute to emotional intelligence, which, in turn, will positively influence teacher-student relationship quality. Consequently, the interplay between mindfulness and teacher-student relationship quality is likely to be partially mediated by emotional intelligence, indicating that both direct and indirect effects will be present in the hypothesized model.

Taken together, the hypothesized model is built on the premise that teacher mindfulness is associated with teacher-student relationship quality, and emotional intelligence plays a mediating role in this association. This research endeavor seeks to augment the current body of knowledge on teacher-student interactions by investigating their associations and potential mediating mechanisms. By shedding light on the importance of nurturing mindfulness and emotional intelligence in educators, the study endeavors to foster positive and nurturing classroom environments. The proposed model is rooted in empirical evidence and established theories, and its validity will be assessed using structural equation modeling to appraise its goodness-of-fit and test the proposed hypotheses.

## Methodology

### Participants

Participants in this research comprised 369 English teachers from diverse schools across three major cities in China. The selection of participants was based on their willingness to participate in the study and their availability during the data collection period. All participants were currently employed as full-time English teachers in either public or private schools, reflecting a mix of urban and suburban settings. Among the participants, there were 158 male English teachers and 211 female English teachers, representing a fairly balanced gender distribution. Their ages ranged from 22 to 55 years, with an average age of 29.62 years, indicating a relatively young and dynamic teaching workforce. The participants boasted varying levels of teaching experience, spanning from 2 to 25 years, with an average of 9.36 years of teaching experience.

The data collection process was conducted in collaboration with school administrators who enthusiastically supported the research endeavor. With their cooperation, access to English teachers was facilitated, and the data collection process was efficiently organized. To maintain the confidentiality and anonymity of the participants, all collected data were securely stored and coded without any personally identifiable information. Before their inclusion in the study, each participant was provided with an informed consent form, detailing the study’s objectives, procedures, potential risks, and benefits. Participants were explicitly informed of their rights as research participants and the voluntary nature of their involvement. In this way, informed consent was obtained from each participant, affirming their willingness to contribute to the study.

### Measures

#### Mindfulness

Mindfulness was evaluated using the Mindfulness Attention Awareness Scale (MAAS), a self-report tool developed by Brown and Ryan (2003). This scale gauges participants’ inclination to be attentive to their present experiences. The MAAS comprises 15 items, and respondents rate their agreement on a 6-point Likert scale, ranging from ‘almost always’ (1) to ‘almost never’ (6). An example item reads, ‘I find it challenging to remain focused on the current moment.’ Previous research (Brown and Ryan, 2003) has established the scale’s sound psychometric properties. The MAAS showed high internal consistency, with a Cronbach’s alpha coefficient of 0.89 in the present investigation. Additionally, McDonald’s omega was calculated and found to be 0.92, further confirming the scale’s reliability.

#### Teacher-child relationship quality

Teacher-child relationship quality was assessed using the Teacher-Student Relationship Scale (STRS; Pianta and Steinberg, 1992). Extensive research in China has validated the scale, confirming its strong reliability and validity (Zhang and Nurmi, 2012; Wang et al., 2023). The STRS comprises 28 items, divided into three sub-scales: closeness, conflict, and dependency. For instance, a sample item reads, “This student openly shares his/her feelings and experiences with me.” Participants rated the extent to which each item described their experiences on a Likert-5 scale, ranging from 1 (definitely not applicable) to 5 (definitely applicable). To derive scores, the items were averaged. Notably, the conflict sub-scale is inversely formulated, implying that a higher score indicates a positive relationship characterized by trust, warmth, and minimal conflict. In this study, the STRS demonstrated satisfactory reliability coefficients, with Cronbach’s alpha values of 0.88 for the overall scale, and 0.82, 0.86, and 0.76 for the closeness, conflict, and dependency sub-scales, respectively. McDonald’s omega for the overall scale was calculated as 0.89, providing additional evidence of the scale’s internal consistency and reliability.

#### Emotional intelligence

Emotional intelligence was evaluated using the Emotional Intelligence Scale (EIS) (Schutte et al., 1998). The survey consisted of 33 items, which were reverse-scored and rated on a 5-point Likert scale, ranging from 1 to 5. Scoring was established with values of 5, 28, and 33. Scores on the scale ranged from 33 to 165, encompassing the potential responses. For this research, the SSEIT with its 33 items was employed, and a fully exploratory approach was adopted to examine the factor structure in the current sample. An example item from the questionnaire reads, “I help other people feel better when they are down.” The EIS demonstrated high internal consistency, with a Cronbach’s alpha of 0.89 in this study. McDonald’s omega was also computed for the EIS, yielding a value of 0.91, further substantiating the scale’s internal consistency and reliability.

### Data collection procedure

Following the selection of schools for the study, a comprehensive list of currently employed English teachers was obtained from the school administrators. From this list, potential participants were identified, and invitations were extended to the English teachers, clearly outlining the study’s purpose and significance. Ensuring the voluntary nature of their participation, teachers were assured that their involvement would have no bearing on their employment status or any other professional aspects, encouraging them to join the study based on their willingness to contribute.

To minimize disruptions to the teachers’ teaching schedules, a suitable data collection period was collaboratively determined in consultation with the school administrators. Throughout the data collection process, stringent measures were implemented to uphold the confidentiality and anonymity of the participants. To ensure this, the questionnaires were coded without any personally identifiable information, safeguarding their privacy. During the data collection phase, the participants were asked to complete three self-report questionnaires. These questionnaires aimed to assess various aspects of their mindfulness, teacher-student relationship quality, and emotional intelligence. To accommodate the preferences of the participants, the questionnaires were made available in both online and paper formats. The online questionnaires were securely distributed through appropriate platforms to ensure the confidentiality of the data. For those who preferred a physical copy, paper questionnaires were distributed in person and subsequently collected.

### Statistical analysis

In this study, we employed a latent variable approach and used parceling to create first-order latent constructs. Parceling involved combining original items to enhance reliability, promote normality, and reduce model parameters, thus minimizing measurement errors (Little et al., 2002). For the Teacher-student relationship quality scale, which consisted of three underlying sub-scales, we used the item-to-construct balance approach to create three parcels (Little et al., 2002).

To address common method bias, we utilized the single-common-method-factor technique (Podsakoff et al., 2003). Descriptive statistics and correlations among the constructs were computed before conducting structural equation modeling (SEM) with AMOS 26.0 to explore the impact of teacher mindfulness on the quality of the teacher-student relationship. Teacher mindfulness served as the predictor variable, while the teacher-student relationship was the criterion variable. Control variables such as teachers’ gender, years of experience, and age were included to account for potential influences.

Additionally, we performed a mediation analysis to examine the relationship between teacher mindfulness (independent variable), the teacher-student relationship (dependent variable), and teacher emotional intelligence as the mediator. Control variables were also incorporated and controlled for in the model. The significance of the mediating effect was assessed using bootstrapping, extracting one thousand samples with repeated random sampling. A mediation effect was considered statistically significant at the 0.05 level if the 95% bootstrap confidence intervals (CIs) did not encompass zero.

Model fit was assessed using four goodness-of-fit measures: the comparative fit index (CFI), the Tucker-Lewis index (TLI), the root mean square error of approximation (RMSEA), and the standardized root mean square residual (SRMR). Adequate model fit was achieved if CFI and TLI scores were above 0.90, RMSEA was below 0.06, and SRMR was below 0.08, following guidelines by Hu and Bentler (1999). Subsequently, to assess the consistency of the mediation effect of emotional intelligence across genders, we conducted multigroup analyses. This analysis examined configural invariance, metric invariance, and scalar invariance (Dimitrov, 2017). If scalar invariance was established, additional analysis was conducted to evaluate the invariance of path coefficients across genders. We used chi-square difference (Δχ^2^) and CFI difference (ΔCFI) as indicators. A substantial Δχ^2^ and a negative ΔCFI value below −0.01 would suggest non-invariance (Cheung and Rensvold, 2002).

## Results

Initially, we applied the single-common-method-factor technique to examine potential common method bias in the self-report scales used to measure three constructs by teachers (namely, mindfulness, EI, and teacher-student relationship) (Podsakoff et al., 2003). In this regard, we compared the hypothesized three-factor model encompassing the three constructs with various alternative models. The results of these comparisons are presented in Table 1. The findings revealed that the hypothesized three-factor model exhibited superior fit to the data (*χ*^2^ (52) = 102.362, CFI = 0.992, TLI = 0.988, RMSEA = 0.011, SRMR = 0.022). Overall, the hypothesized three-factor model demonstrated significantly superior fit compared to all four competing models. These findings suggest that the presence of common method bias had negligible impact on the results.

**Table 1:** Results of model fit for the common method bias test.

Models	χ2	df	CFI	TLI	RMSEA	SRMR
M1: Single-factor model	450.120	56	0.675	0.640	0.138	0.98
M2: Two-factor model 1	365.890	55	0.811	0.778	0.123	0.089
M3: Two-factor model 2	232.161	54	0.926	0.889	0.071	0.055
M4: Two-factor model 3	268.705	55	0.910	0.885	0.072	0.067
M5: Hypothesized three-factor model	102.362	52	0.992	0.988	0.011	0.022

All competing models were compared with the hypothesized three-factor model. M2: two-factor model (mindfulness and teacher-student relationship are combined); M3: two-factor model (EI and teacher-student relationship are combined); M4: two-factor model (mindfulness and EI are combined); ^***^p < 0.001.

Also, to ensure the appropriateness of the statistical analyses, measures of distribution normality were examined for each variable. This included assessments of skewness and kurtosis, along with corresponding standard errors for skewness and kurtosis. The results of these analyses are summarized in Table 2.

**Table 2:** Distribution normality assessment.

Variable	Skewness	Standard error (skewness)	Kurtosis	Standard error (kurtosis)
Mindfulness	−0.21	0.11	0.23	0.14
EI	0.19	0.09	0.19	0.09
Teacher-Student Relationship	−0.13	0.06	0.17	0.08

As demonstrated in Table 2, the distribution normalities for the variables of interest fall within an acceptable range, showing that the data are generally close to normal, with slight deviations in skewness and kurtosis values.

Table 3 presents the descriptive statistics and correlations among the variables of interest. The correlation between mindfulness and emotional intelligence was found to be positive and statistically significant at the *p* < 0.05 level (*r* = 0.29). Additionally, there was a significant positive correlation between mindfulness and teacher-student relationship quality (*r* = 0.48, *p* < 0.01), as well as a significant positive correlation between emotional intelligence and teacher-student relationship quality (*r* = 0.39, *p* < 0.01). The results suggest that mindfulness is positively associated with both emotional intelligence and teacher-student relationship quality. Furthermore, emotional intelligence also demonstrated a positive correlation with teacher-student relationship quality.

**Table 3:** Descriptive statistics and correlations.

	*M* (SD)	1	2	3
(1) Mindfulness	3.71 (0.91)	1.00		
(2) EI	3.16 (0.79)	0.29*	1.00	
(3) Teacher-student relationship	3.46 (0.88)	0.48**	0.39**	1.00

*p < 0.05. **p < 0.01.

**Figure 1:**
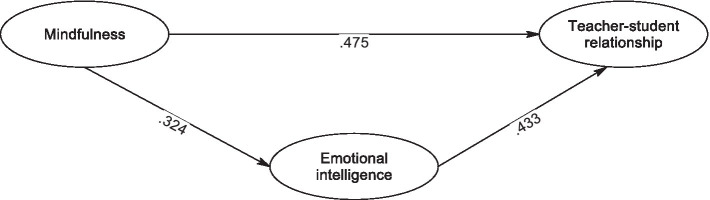
The fit model. All path coefficients were significant at *p* < 0.01.

Next, we computed the overall impact of teacher mindfulness on the quality of the teacher-student relationship. The findings revealed a well-fitted model (*χ*^2^ (86) = 171.252, *p* < 0.001, CFI = 0.952, TLI = 0.943, RMSEA = 0.042, SRMR = 0.034) and a significant total effect (β = 0.436, SE = 0.04, *p* < 0.01). Moving on to investigate the mediating role of teacher emotional intelligence, the model fit indices demonstrated a good fit (*χ*^2^ (198) = 179.825, *p* < 0.001, CFI = 0.957, TLI = 0.951, RMSEA = 0.039, SRMR = 0.033). Figure 1 presents the path coefficients. The outcomes indicated a positive and significant association between teacher mindfulness and teachers’ emotional intelligence (β = 0.324, SE = 0.07, *p* < 0.01), and teachers’ emotional intelligence significantly influenced the teacher-student relationship (β = 0.433, SE = 0.09, *p* = 0.01). The mediating effect of teachers’ buoyancy was found to be significant (β = 0.140, 95% CI = [0.07, 0.22]). Furthermore, the direct effect of mindfulness on the teacher-student relationship remained significant (β = 0.475, SE = 0.11, *p* = 0.01), indicating partial mediation.

Subsequently, to examine the mediating role of teacher emotional intelligence and its consistency across teachers’ genders, a series of multigroup analyses were conducted. Initially, separate baseline models were tested for each gender, and the findings indicated a favorable fit to the data (refer to Ma and Mb in Table 4). Following that, configural invariance was assessed, and the results demonstrated a satisfactory fit to the data (refer to Mc in Table 4). Additionally, metric invariance was evaluated by equating factor loadings across the groups (see Md). The results revealed that the model exhibited a good fit, and the difference between Md and Mc was not statistically significant (Δ*χ*^2^ (1) = 18.959, *p* = 0.41, ΔCFI = 0.001), providing evidence of metric invariance.

**Table 4:** Results of fit indices for gender invariance.

Model	*χ*^2^ (*df*)	CFI	TLI	RMSEA	SRMR	Δ*χ*^2^ (Δ*df*)	*p*	ΔCFI
Ma: Baseline—Female	345.142 (181)^**^	0.936	0.927	0.045	0.046	–	–	–
Mb: Baseline—Male	327.106 (181) ^**^	0.958	0.951	0.036	0.047	–	–	–
Mc: Configural invariance	547.345 (288)^**^	0.955	0.939	0.042	0.045	–	–	–
Md: Metric invariance	566.304 (289)^**^	0.954	0.936	0.042	0.048	18.959 (1)	0.36	0.001
Me: Scalar invariance	573.456 (296)^**^	0.954	0.937	0.041	0.048	7.152 (7)	0.39	0.000

^**^p < 0.01.

Moving on to the fourth step, we conducted an assessment of scalar invariance. In this examination, item intercepts were equated across the groups, leading to a well-fitted model and no significant difference between this model (Me) and the previous one (Md), as evidenced by the non-significant change in chi-square (Δ*χ*^2^ (7) = 7.152, *p* = 0.45, ΔCFI = 0.000). These results provide confirmation of scalar invariance.

## Discussion

In this research, we examined the association between teacher mindfulness and the quality of teacher-student relationships, with a particular focus on the mediating influence of emotional intelligence. The findings shed light on the importance of mindfulness and emotional intelligence in the context of teacher-student interactions and provide valuable insights for educational practices and interventions.

Firstly, our study confirms a significant and positive correlation between teacher trait mindfulness and the quality of teacher-student relationships, supporting *hypothesis 1*. This aligns with previous research (Becker et al., 2017; Lemberger-Truelove et al., 2018; Braun et al., 2019; Moyano et al., 2021; Wang et al., 2023). Instructors who exhibit higher levels of attentional ease, sustained focus, and an acceptance of the present moment report closer, more emotionally fulfilling, less stressful, and less strained relationships with their students (Becker et al., 2017). This suggests that a mindful disposition equips instructors with a more objective perspective when facing challenging interactions with students. Additionally, mindfulness practice appears to mitigate difficulties in teacher-student relationships (Emerson et al., 2017). Consistent with Lemberger-Truelove et al. (2018), heightened levels of mindfulness among teachers translate into an enhanced ability to evaluate individuals’ behaviors impartially and objectively. This is especially prominent when demonstrating tolerance and understanding towards students’ challenging behaviors, ultimately nurturing stronger bonds between instructors and pupils.

Teacher-student relationships are dynamic, mutually influential, and multifaceted. The extent of mutual affinity and the frequency of interaction play pivotal roles in shaping this connection. Educators with elevated levels of mindfulness exhibit a tendency to approach conflicts and student errors with greater acceptance and pragmatism, thereby fostering a positive feedback loop in the teacher-student relationship. Through mindfulness practices, individuals develop the capacity to be fully present and attentive in their interactions, leading to more profound connections and a heightened capacity for empathetic understanding (Roeser et al., 2012). Consequently, teachers who demonstrate heightened mindfulness are likely to possess a heightened awareness of their students’ emotional needs, coupled with an increased sensitivity to their experiences, enabling them to respond adeptly to their challenges (MacDonald and Shirley, 2009). This equips them to establish and nurture positive, supportive, and trusting relationships with their students.

In our study, we discovered that teacher mindfulness not only directly predicts teacher-student relationship quality but also operates through the mediation of emotional intelligence, supporting *hypothesis 2*. This finding aligns with prior research (Miao et al., 2018; Mestre et al., 2019; Wang et al., 2023), which emphasizes the significant influence of mindfulness on emotional intelligence. Teachers who practice mindfulness demonstrate a greater capacity to manage their emotions effortlessly, experience emotions without judgment, and foster the growth of emotional intelligence in their students. Moreover, emotional intelligence significantly influences the interactions between instructors and learners. Mindfulness promotes an empathetic and accepting mindset, requiring similar expertise as emotional intelligence. Mindfulness practices, which enhance self-awareness and emotional regulation, have the potential to enhance emotional intelligence by increasing individuals’ sensitivity to their own emotions and the emotions of others (Poulou, 2017; Chamizo-Nieto et al., 2021). Consequently, teachers with higher levels of mindfulness are more likely to develop greater emotional intelligence as they become more proficient in recognizing and managing their own emotions and understanding their students’ emotional needs. In turn, instructors with high emotional intelligence can recognize and control their own emotions and those of their students, facilitating positive interpersonal interactions. Mindful teachers with higher emotional intelligence may skillfully recognize and appropriately respond to their students’ emotional cues and needs (Brandsma, 2017; Chamizo-Nieto et al., 2021). They exhibit greater empathy, understanding, and compassion towards their students, positively influencing the quality of teacher-student interactions.

Teachers with higher levels of emotional intelligence possess a valuable skill set for effectively managing challenging situations and conflicts that may arise in the classroom (Mérida-López and Extremera, 2017). Their ability to navigate these scenarios is enhanced by their skillful management of emotions and their understanding of their students’ emotional needs, ultimately fostering a positive learning environment and promoting strong teacher-student relationships (Geraci et al., 2023; Wang et al., 2023). Moreover, such teachers often cultivate a relaxed and joyful emotional atmosphere within the school, creating a comfortable learning environment for their students. Positive connections between students and teachers, regardless of age, are highly valued, contributing significantly to the quality of the teacher-student relationship. Furthermore, a high degree of emotional intelligence equips instructors to efficiently address a variety of student issues and provide substantial emotional support (Valente and Lourenço, 2020), ultimately contributing to the development of robust teacher-student relationships.

Overall, this research underscores the pivotal role of mindfulness and emotional intelligence in shaping teacher-student relationships. The study reveals a significant positive correlation between teacher trait mindfulness and the quality of these relationships, in line with prior research. Mindfulness equips educators with the ability to approach conflicts and challenges with greater acceptance and objectivity, fostering deeper connections. Furthermore, mindfulness enhances teachers’ awareness of their students’ emotional needs, enabling them to respond adeptly to challenges and nurture supportive relationships. Importantly, the study also uncovers that teacher mindfulness operates through the mediation of emotional intelligence, highlighting the interplay between these constructs. Mindfulness practices enhance emotional intelligence by increasing individuals’ sensitivity to emotions, ultimately influencing positive interactions. Educators with higher emotional intelligence exhibit adeptness in managing classroom situations, creating a conducive learning environment, and forming meaningful connections with their students. This research provides valuable insights for educational practices, emphasizing the significance of fostering mindfulness and emotional intelligence in teacher-student dynamics.

Although this study provides valuable insights into the synergistic relationship between teacher mindfulness and emotional intelligence in shaping teacher-student relationships, it is imperative to acknowledge certain limitations inherent in the research design. These limitations offer avenues for future research to expand and refine our understanding of these constructs. First and foremost, the study’s reliance on a convenience sampling method is acknowledged as a limitation. This approach, while practical for the current study, may reduce the generalizability of our findings beyond the sampled population. In future research endeavors, employing a probabilistic sampling technique would offer greater scope for data generalization across broader educational contexts.

Additionally, the cross-sectional nature of the study design poses a limitation. This temporal snapshot restricts our ability to establish causal relationships definitively. Longitudinal studies could provide a more nuanced exploration of the temporal sequences and clarify the direction of causality between teacher mindfulness, emotional intelligence, and teacher-student relationship quality. Furthermore, the use of self-report measures, though a common approach, introduces potential biases including social desirability and common method variance. Future research would benefit from incorporating objective measures and multiple data sources to enhance the robustness and validity of our findings. The exclusive focus on Chinese English teachers in this study may also constrain the generalizability of our results to other cultural contexts and subject areas. It is recognized that cultural nuances can significantly impact the expression and perception of mindfulness, emotional intelligence, and teacher-student relationships. To address this, future studies could include a more diverse sample representing a broader range of cultural backgrounds and educational disciplines, enabling a more comprehensive examination of cross-cultural applicability.

Additionally, this study did not delve into potential alternative mediators or moderators that may influence the relationship between teacher mindfulness and teacher-student relationship quality. Future research endeavors could explore additional psychological and contextual factors to gain a more comprehensive understanding of this intricate relationship. Finally, while the sample size was determined to be adequate for the analyses conducted, it is acknowledged that it may limit the statistical power to detect small effect sizes and potentially impact the generalizability of our findings. Therefore, replication studies with larger and more diverse samples would bolster the robustness and external validity of the results. In light of these limitations, we view them as opportunities for future research to further refine and broaden our comprehension of the interplay between teacher mindfulness, emotional intelligence, and teacher-student relationships. Addressing these areas will undoubtedly contribute to a more nuanced and comprehensive understanding of effective teacher-student dynamics in diverse educational settings.

## Conclusion

This study provides valuable insights into the relationship between teacher mindfulness and teacher-student relationship quality, with emotional intelligence playing a mediating role in this dynamic. We found strong evidence supporting the direct positive relationship between teacher mindfulness and teacher-student relationship quality. The significant direct effect observed in this study suggests that teachers who exhibit higher levels of mindfulness are more likely to foster positive and supportive relationships with their students. Mindfulness practices, which encourage present-moment awareness and non-judgmental acceptance, may enable teachers to be more attuned to their students’ needs, emotions, and experiences, leading to enhanced teacher-student interactions and a more positive classroom climate. Importantly, our findings also reveal that emotional intelligence serves as a critical mediator in this relationship. Teachers who possess higher emotional intelligence may be better equipped to navigate the complexities of the teacher-student dynamic by effectively recognizing and managing emotions, both in themselves and in their students. Emotional intelligence can facilitate empathy, understanding, and effective communication, which are crucial for establishing and maintaining positive teacher-student relationships.

The findings of this study hold practical significance for both educators and school administrators. One key implication is that educational institutions should consider incorporating mindfulness training and practices into teacher professional development programs. By fostering mindfulness among teachers, institutions can empower them to be more sensitive and attuned to their students’ emotional and behavioral needs. This heightened awareness can, in turn, lead to the cultivation of more positive, nurturing, and supportive teacher-student relationships. Furthermore, the study highlights the pivotal role of emotional intelligence as a mediating factor in the relationship between teacher mindfulness and the quality of teacher-student relationships. This underscores the importance of nurturing emotional intelligence among educators. To achieve this, educational institutions can integrate emotional intelligence training into teacher education programs. Such training equips teachers with the tools and knowledge to better understand, manage, and express their own emotions and, crucially, to empathize with their students’ emotions. This enhancement of emotional intelligence can contribute to the establishment of teacher-student relationships characterized by emotional responsiveness and empathy, which are fundamental for creating a positive and supportive learning environment.

Investing in the enhancement of teacher-student interactions can have a profound impact on the overall well-being of educators. Specifically, fostering intimacy and reducing conflict in these relationships can lead to more genuine emotional expression. This means that teachers can feel more comfortable expressing their true emotions and experiences, without resorting to pretense, particularly when trying to shield students they perceive as vulnerable. Such an approach is not only beneficial for teachers’ own happiness but also for the authentic and nurturing atmosphere it creates in the classroom. Moreover, the development of programs that emphasize an organizational commitment to emotional and social development offers a unique opportunity to address achievement disparities, particularly among students living in poverty when compared to their more affluent peers. These programs, rooted in mindfulness-based strategies (as proposed by Zhai et al., 2021), can significantly enhance teacher well-being and teacher-student relationships. For example, educational institutions can encourage teachers to engage in community-based courses that teach mindfulness-based techniques like meditation and yoga. Furthermore, integrating education in these techniques as a part of continuous professional development for teachers and pre-service teacher training can be immensely beneficial. Through these strategies, teachers can experience improved well-being, heightened focus, enhanced emotional regulation, and stronger interpersonal relationships. These positive changes can have a cascading effect, ultimately influencing the emotional environment of the entire school.

## Data availability statement

The raw data supporting the conclusions of this article will be made available by the authors, without undue reservation. Requests to access these datasets should be directed to XW, wxuelucy@hotmail.com.

## Ethics statement

The studies involving humans were approved by Department of Foreign Languages, Henan Finance University, Zhengzhou, Henan, China. The studies were conducted in accordance with the local legislation and institutional requirements. The participants provided their written informed consent to participate in this study.

## Author contributions

XW: Conceptualization, Data curation, Formal analysis, Funding acquisition, Investigation, Methodology, Project administration, Resources, Software, Supervision, Validation, Visualization, Writing – original draft, Writing – review & editing.
